# A Comparative Evaluation of Marginal Leakage of Different Restorative Materials in Deciduous Molars: An *in vitro* Study

**DOI:** 10.5005/jp-journals-10005-1145

**Published:** 2012-08-08

**Authors:** Gunjan Yadav, Usha Rehani, Vivek Rana

**Affiliations:** Senior Lecturer, Department of Pedodontics, Sardar Patel Post Graduate Institute of Dental and Medical Sciences, Lucknow, Uttar Pradesh, India, e-mail: gunjanyadav@rediffmail.com; Ex-Professor and Head, Department of Pedodontics and Preventive Dentistry, Subharti Dental College, Meerut, Uttar Pradesh, India; Associate Professor, Department of Pedodontics and Preventive Dentistry, Subharti Dental College, Meerut, Uttar Pradesh, India

**Keywords:** Colored compomer, Ormocer, Giomer, RMGIC and microleakage

## Abstract

**Context:** Microleakage around dental restorative materials is a major problem in clinical dentistry. Inspite of many new restorative materials available in the market very few actually bond to the tooth surface.

**Aims:** The aims of this study were: (1) To evaluate and compare the marginal leakage of newer restorative materials viz colored compomer, ormocer, giomer and RMGIC in class I restoration of deciduous molars. (2) To compare the microleakage scores between the groups of: Colored compomer and ormocer, giomer and RMGIC, ormocer with giomer and RMGIC, giomer with RMGIC.

**Materials and methods:** A total of 40 primary molars were randomly divided into four groups of 10 each. Class I cavities were prepared and the cavities were restored with colored compomer (Group A), Ormocer (Group B), Giomer (Group C) and RMGIC (Group D). The teeth were thermocycled and subjected to 0.5% basic fuchsin dye penetration followed by sectioning. The cut sections were evaluated under a stereomicroscope and the data was subjected to statistical analysis.

**Statistical analysis used:** Mann-Whitney U test and Student t-test.

**Results:** No significant difference was observed when colored compomer was compared to ormocer, giomer and RMGIC. Ormocer showed significantly lower microleakage when compared to giomer. However, no significant difference was observed when ormocer was compared to RMGIC. No significant difference between giomer and RMGIC was found.

**Conclusion:** Ormocer has proven to be an excellent restorative material as it showed least microleakage followed by colored compomer, giomer and RMGIC in increasing order.

**How to cite this article:** Yadav G, Rehani U, Rana V. A Comparative Evaluation of Marginal Leakage of Different Restorative Materials in Deciduous Molars: An *in vitro* Study . Int J Clin Pediatr Dent 2012;5(2):101-107.

## INTRODUCTION

The goal of restorative dentistry is undoubtedly to restore the tooth to its form and function. One of the requisites is to adapt well and seal the cavity walls for the longevity of the restoration. There has always been a keen interest in the adaptation of dental restorative materials to the walls of the cavity and the retentive ability of a material to seal the cavity against ingress of oral fluids and microorganisms.^1^ Microleakage around dental restorative materials is a major problem in clinical dentistry. It may be defined as ‘the clinically undetectable passage of bacteria, fluids, molecules or ions between a cavity wall and the restorative materials applied to it'.^2^ This seepage can cause hypersensitivity of restored teeth, tooth discoloration, recurrent caries, pulpal injury and accelerated deterioration of some restorative materials.^1^

Inspite of many new materials in the market like GIC, composite, compomers, giomers and now ceromers and ormocers very few materials actually bond to the tooth surface. Dimensional changes and lack of adaptation of the restoration to cavity walls can lead to marginal leakage with fluid and molecular movement and the ingress of bacteria or bacterial nutrients.^3^ Thus, the procurement of a perfect seal on the restoration tooth interface is one of the prime goals of restorative dentistry in order to prevent penetration of microorganisms and other contaminants. Thus, it is evident that poor marginal seal impacts a major drawback in longevity of restoration.

Fluoride releasing and chemical bonding properties of glass ionomer cements are well known. However, poor physical properties, such as tendency to undergo surface crazing, low fracture resistance and esthetics limit its use.^1^ To overcome this resin-modified glass ionomers (RMGIs) were developed. The advantages of RMGIs are that it is biocompatible and fluoride release is similar to those of conventional GI, improved physical properties, especially with regard to tensile strength and abrasion resistance, better wear resistance, good adhesion to enamel and dentin and satisfactory esthetics.^4^

Because of perceived inadequacies in the ease of use of composites as several clinical steps are required to obtain a good interfacial bond, compomers were introduced. Colored compomers have been introduced in the market in various attractive colors with glitter effect. It offers a perfect solution for nervous, frightened and impatient children. It shows excellent physical properties and high fluoride release for prevention of secondary caries.^5,6^

The research of finding a filling material which was superior to contemporary composite has led to the evolution of a new material called ormocers. Ormocer–the acronym of organically modified ceramic is a new material for all filling indications in the anterior and posterior area which serve as an optimum and up to date replacement for amalgam, composites and compomers.^7-9^

In the continuing quest for improved glass ionomer like restorations manufacturers have introduced a new class of materials called giomers. It has properties of both glass ionomers (fluoride release and fluoride recharge) and resin composites–(excellent esthetics, easy polishability and biocompatibility).

Thus, it is evident from the literature and long experiences of dental professionals that poor marginal seal imparts a major drawback in longevity of the restoration. So, the present *in vitro* study is envisaged to compare the marginal leakage of colored compomer, ormocer, giomer and resin-modified glass ionomer cement in class I restoration of deciduous molars.

## MATERIALS AND METHODS

The present *in vitro* study was carried out in the Department of Pedodontics and Preventive Dentistry, Subharti Dental College, Meerut in collaboration with Department of Endocrinology, Central Drug Research Institute, Lucknow and Birbal Sahni Institute of Paleobotany, Lucknow to evaluate and compare the marginal leakage of various restorative materials in deciduous molars.

A total of 40 noncarious primary molars which were extracted for the reason of overretention were selected for the study. Surface debridement of all the teeth was done with hand instruments and the teeth were stored in normal saline at room temperature till further use. The teeth were randomly divided into four groups of 10 teeth each.

Standard class I cavities were prepared on all the 40 teeth using round bur, straight bur and inverted cone bur with a low speed contra angle hand piece using constant water-spray. The depth of the cavity was standardized to 1 to 2 mm with no mechanical retention form. Prepared cavities were then thoroughly cleaned with water and gently dried before the placement of the restoration.

**Fig. 1:**
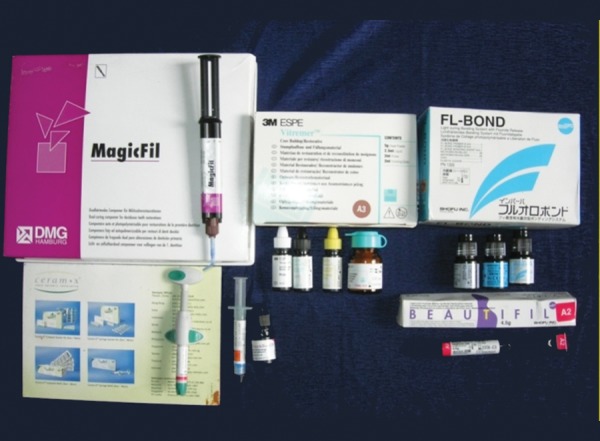
Restorative materials

**Fig. 2:**
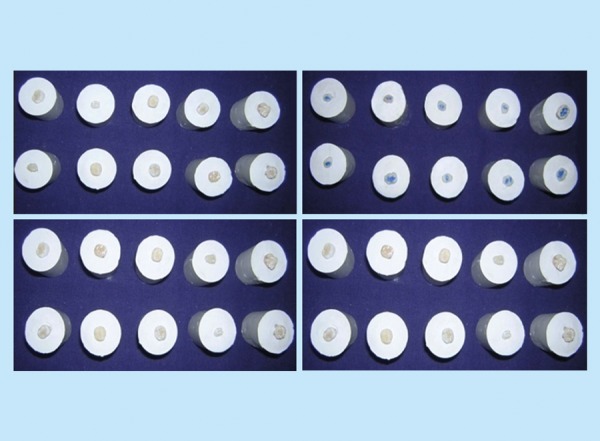
Samples restored with different materials

In each group the cavity was restored with its respective restorative material according to the manufacturer's instructions (Figs 1 and 2).

**Table:** 

*Groups*		*Restorative material*		*Commercial name*		*Manufacturing company*	
Group A		Colored compomer		MagicFil		Zenith/DMG, Englewood,NJ	
Group B		Ormocer		Ceram X		Dentsply	
Group C		Giomer		Beautifil		Shofu Inc Kyoto Japan	
Group D		Resin modified glass ionomer cement		Vitremer		3M Dental product, USA	

After 24 hours of storage in distilled water, teeth were then subjected to thermocycling for 200 cycles at a temperature of 4°C ± 2°C and 50°C ± 2°C with dwell time of 10 seconds.

**Fig. 3:**
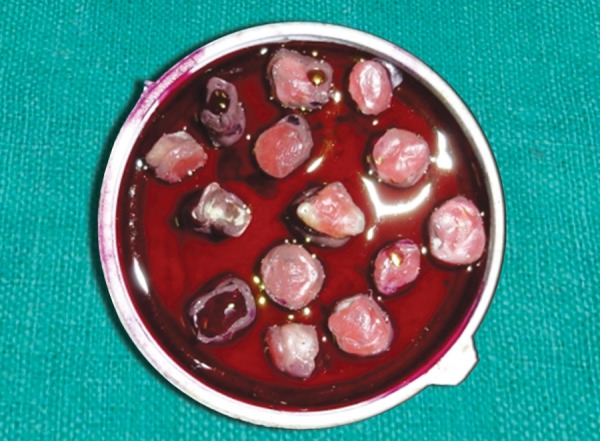
Samples following nail varnish application and dye penetration

**Fig. 4:**
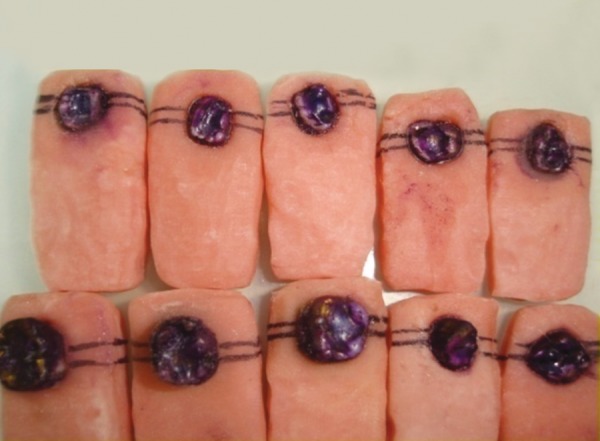
Sample preparation for section cutting

**Fig. 5:**
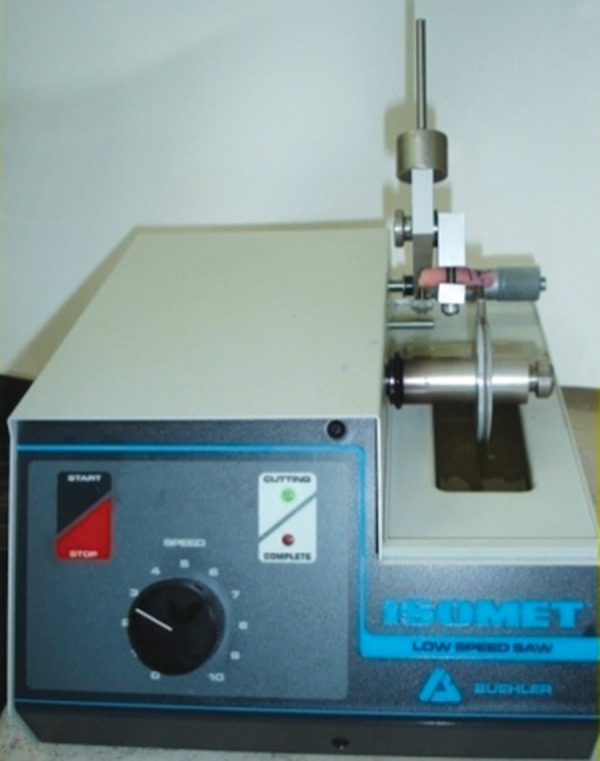
Low speed diamond saw

The specimens were then prepared for dye exposure. Each tooth was covered with nail polish except an area approximately within 1 mm of periphery of the restoration. The apices of the teeth were occluded with modeling wax to prevent leakage through root apices. The teeth were then immersed in 0.5% basic fuchsin dye for 24 hours at room temperature (Fig. 3). After removal from the dye, samples were thoroughly cleaned and rinsed under tap water until all the dye was removed from the surface. Then the samples were mounted in self-curing acrylic blocks (Fig. 4) and sectioned buccolingually through the center of the restoration with a low-speed diamond saw (Fig. 5).

**Fig. 6:**
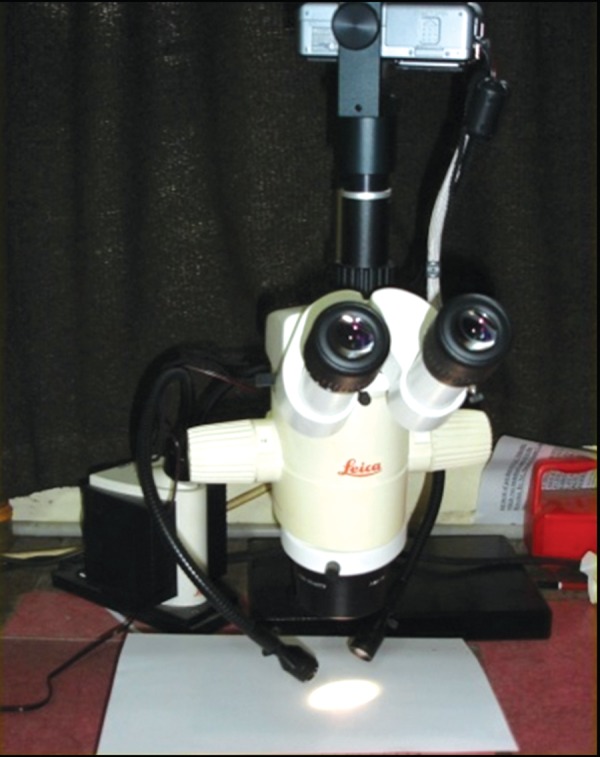
Stereomicroscope used to check microleakage in the specimen

The teeth were then examined under stereomicroscope (16X magnification) to measure the depth of the dye penetration at the two surfaces of the cavity and the score which was higher was given as a score to the particular tooth (Fig. 6). All the scoring was carried out by a single person and the scoring criteria used for the study was as follows:

The following criteria were used to score dye penetration.^10^

**Table:** 

0		No dye penetration (Fig. 7)	
1		Dye penetration between the restoration and the tooth into enamel only (Fig. 8)	
2		Dye penetration between the restoration and the tooth into enamel and dentin (Fig. 9)	
3		Dye penetration between the restoration and the tooth into the pulp chamber (Fig. 10)	

The score was tabulated, interpreted and the resultant findings were statistically analyzed.

## RESULTS

All the 40 samples were evaluated; score of dye penetration, mean and standard deviation were calculated. Mann- Whitney U-test (Table 2) and Student t-test were used for the comparison between the groups.

**Fig. 7:**
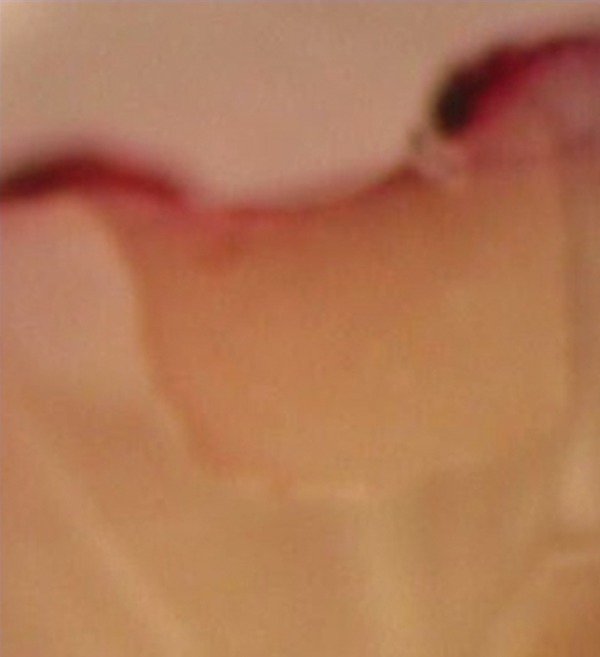
No dye penetration (score 0) in Ormocer

**Graph 1:**
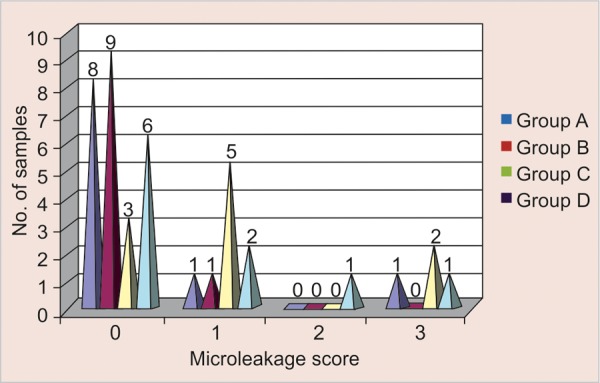
Descriptive statistics of the comparison of microleakage scores among various restorative materials

Table 1 and Graph 1 show that out of the 10 teeth filled with colored compomer eight teeth showed no dye penetration at all, one tooth showed dye penetration up to enamel, i.e. score 1, and one tooth showed dye penetration into the pulp chamber (score 3).

Of the 10 teeth filled with ormocer, nine teeth showed no dye penetration, and one tooth showed penetration with score 1.

Of the 10 teeth filled with giomer, three teeth showed no dye penetration, five showed score of one, another two showed microleakage with score 3.

**Table 1:** **Table 1:** Descriptive statistics of comparison of microleakage scores among various restorative materials

*Restorative materials*		*No. of samples*		*Microleakage scores*								*Mean score*		*SD*		*Mean score ± SD*	
				*0*		*1*		*2*		*3*							
Colored compomer (group A)		10		8		1		_		1		0.4		0.9661		(0.4 ± 0.9661)	
Ormocer (group B)		10		9		1		_		_		0.1		0.3162		(0.1 ± 0.3162)	
Giomer (group C)		10		3		5		_		2		1.1		1.1005		(1.1 ± 1.1005)	
RMGIC (group D)		10		6		2		1		1		0.7		1.0594		(0.7 ± 1.0594)	

Of the 10 teeth filled with RMGIC, six teeth showed no dye penetration, two showed score of 1, another one tooth showed microleakage with score 2 and one tooth showed microleakage upto the level of pulp chamber, i.e. score 3.

Ormocer showed the least microleakage with mean score and standard deviation of 0.1 ± 0.3162 as compared to colored compomer with mean score and standard deviation of 0.4 ± 0.9661 and RMGIC with mean score and standard deviation of 1.1 ± 1.1005.

No significant difference was observed when colored compomer was compared to ormocer, giomer and RMGIC.

Ormocer showed significantly lower microleakage (p < 0.05) when compared to giomer. However, no significant difference was observed when ormocer was compared to RMGIC.

There was no significant difference when giomer was compared to RMGIC.

## DISCUSSION

A major goal in restorative dentistry is the control of marginal leakage which may occur because of dimensional changes or lack of adaptation of the restorative materials to the cavity preparation. These interfacial gaps may lead to recurrent caries and pulpal pathosis.^11^

It is apparent that microleakage around restorations is a series of phenomena and not a single entity. Although ionic charge and chemical reactivity of diffusing fluids have a part in marginal leakage, the physical and chemical nature of restorative materials and the clinical skills of the operator play equally important roles. It must be recognized that application of the restorative materials *in vivo* is more difficult than their application *in vitro* on extracted teeth. An adequate seal *in vivo* is unquestionably and undoubtedly difficult to obtain.

In the present study, microleakage was seen to some extent with almost all the dental restorative materials. This has been suggested earlier by Gladys S et al (2001)^12^ that microleakage can be expected with all the dental restorative materials developed till date. In our study, we found that the least microleakage occurred around the ormocer group and the maximum microleakage was seen in giomer group. Microleakage observed in various groups can be summarized as: Group B (ormocer) < group A (colored compomer) < group D (RMGIC) < group C (giomer).

Our findings are in agreement with the findings of Yazici AR et al (2003)^13^ who studied the microleakage of class V cavities restored with three different types of flowable resin restorative material. They reported that ormocer was more effective than flowable composite and flowable compomer. The reason suggested for this was that ormocers have an inorganic backbone based on silicon dioxide and are functionalized with polymerizable organic units to produce three-dimensional compound polymer.

**Table 2:** **Table 2:** Descriptive statistics of comparision of microleakage in different groups using two sample rank test (Mann-Whitney U-test)

*Groups*		*U*		*p*		*Result*	
Group A *vs* group B		U = 44.50		0.684		Microleakage score is higher in group A than group B	
Group A *vs* group C		U = 26.50		0.075		Microleakage score is higher in group C than group A	
Group A *vs* group D		U = 40.50		0.481		Microleakage score is higher in group D than group A	
Group B *vs* group C		U = 19.00		0.019		Microleakage score is significantly higher in group C than group B	
Group B *vs* group D		U = 34.00		0.247		Microleakage score is higher in group D than group B	
Group C *vs* group D		U = 37.00		0.353		Microleakage score is higher in group C than group D	

Standard (0.05, 9) = 23.01, standard p < 0.05 = significant, U < 23 = significant

**Fig. 8:**
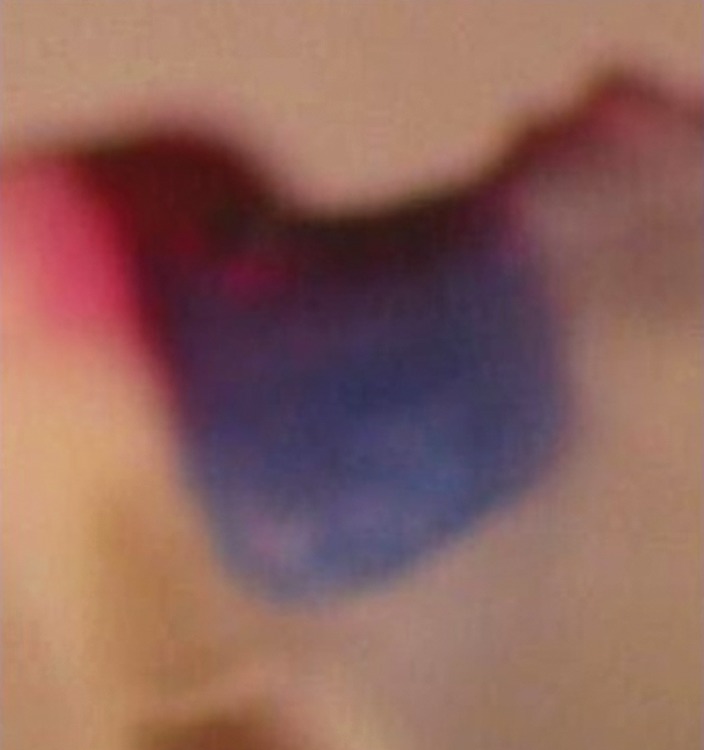
Teeth with dye penetration (score 1) in colored compomer

**Fig. 9:**
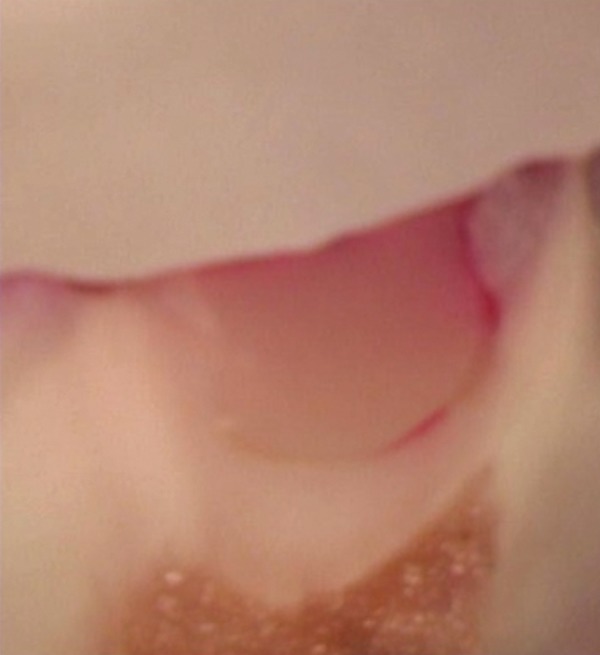
Teeth with dye penetration (score 2) in RMGIC

**Fig. 10:**
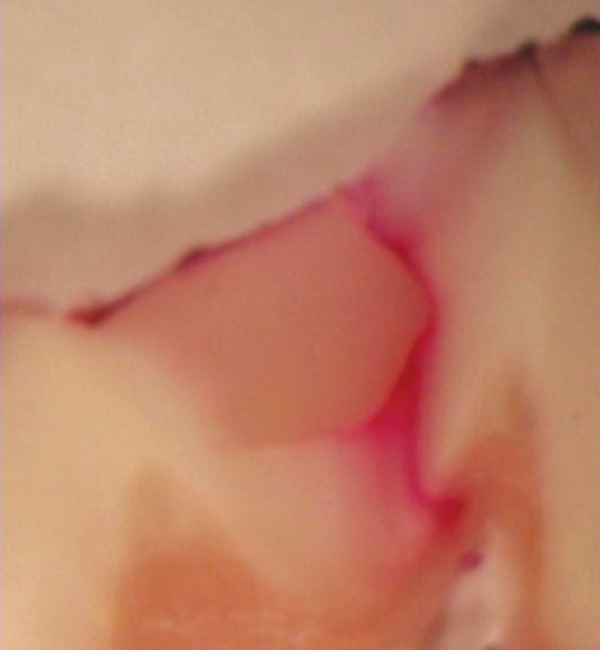
Teeth with dye penetration (score 3) in giomer

The probable reason for decreased microleakage in ormocer group may be due to its structure which is a bio- compatible polysiloxane net with low shrinkage even prior to light curing. The inorganic network formation starts by hydrolysis and proceeds by polycondensation of Si(OR)_3_ groups. Starting with silane, polysiloxanes with polymersiable groups are formed. Ormocers are fully polymerized due to their preformed structure and their extremely high molecular weight, so they undergo considerably less shrinkage than composites or compomers. A possible explanation for the lower microleakage scores may be the three-dimensional structure and low modulus of elasticity which may have reduced polymerization shrinkage.^13^ Further, Hickel R et al (1998)^9^ and Jain P et al (2001)^7^ reported that reduced shrinkage, results in requiring less adhesion power of the adhesive and especially in the long run less marginal gap is expected.

Schirremeister JF et al (2004)^14^ reported that ormocer (Ceram X) was clinically effective in combination with prime and Bond XP as no recurrent caries was recorded and none of the restorations caused sensitivity after 6 months and the marginal integrity also remained grade A for 36 restorations out of 38. Similar result was seen in our study, in which using Prime and Bond XP, the marginal sealing ability of ormocer ranked higher than the other restorative materials.

In the present study compomer had comparatively decreased marginal leakage which is similar to the studies reported earlier by Morabito A et al (1997),^11^ Welbury RR et al (2000),^15^ Sikri V et al (2002).^16^ Mali P et al (2006)^1^ compared the microleakage of glass ionomer, composite resin and compomers. They concluded that microleakage was evident in all restorative materials, with glass ionomer showing maximum leakage followed by composite and compomers demonstrated the best results with minimum leakage. The results obtained in the study are similar to that of ours.

The leakage in compomer was less than RMGIC and giomer because of unique dual cure feature of colored compomer. Other compomers undergo resin polymerization only by light exposure. The two-component colored compomer is blended thoroughly while being injected through the mixing tip of the double-barreled syringe, thus initiating chemical polymerization. Self-curing gives assurance that material hardening will occur within 5 minutes throughout the resin mass, even if radiant energy from the light beam fails to penetrate completely.^5^

Contradictory results to our study have been reported earlier by Toledano et al (1999).^17^ They reported that microleakage was significantly higher with compomer when compared with resin-modified glass ionomer whereas Rodrigus JA et al (1999)^18^ stated that microleakage patterns of composite were similar to that of the compomers. Bracket et al (1998)^19^ concluded that there was no significant difference in microleakage between two lightcured glass ionomer restorative materials and a compomer, this differed from the conclusion we found in our study as in our study there was a slight significant difference between the two groups.

In our study RMGIC had greater microleakage score than ormocer and compomer which could be due to the immediate finishing/polishing procedure which was employed in our study according to the manufacturer's instructions. It has been reported by Yap AUJ et al (2002)^20^ that in addition to surface roughness, immediate finishing/ polishing could compromise the marginal seal of RMGIC to tooth. Although immediate finishing/polishing did not effect the marginal seal to dentin, it increased microleakage at enamel margins.^20^ Similar conclusions were drawn by Irie and Suzuki 1999^21^ and 2002.^22^

In our study the microleakage score of the giomer restorative material is the highest thereby suggesting the other three materials to be better than giomer with regards to the marginal integrity. Since, there were several steps involved in our study like storage, thermocycling, manipulation, dye penetration, etc. so the result can be due to any variation in any of the above procedures. As giomer is a new material and not much of studies have been done on it, so more work is needed to be done to have a better opinion of this new material. In *in vitro* studies, as ours, the teeth were not subjected to mechanical stress, occlusal wear and other biological factors. Hence, long-term stability of the restorative material *in vivo* and marginal sealing ability of such restorations should be investigated in order to have a better option for restorations in primary molars.

Although the results obtained from the present study may not be correlated to clinical situations, nevertheless they provide some useful information regarding these latest restorative materials.

## SUMMARY AND CONCLUSION

The following conclusions can be drawn from the present study:

1. The marginal sealing ability of ormocer was found highest among all the dental restorative materials used in the study. The giomer restorative material expressed the lowest marginal sealing ability.
2. The microleakage scores of colored compomer, ormocer, giomer and RMGIC can be summarized as: Ormocer < colored compomer < resin modified glass ionomer cement < giomer.

### Note

Ormocer has proven to be an excellent restorative material as it showed least microleakage followed by colored compomer, giomer and RMGIC in increasing order. As giomer is a new material and not much of studies have been done on it so more work is needed to be done to have a better opinion of this new material.
